# Association of Gestational Free and Total Triiodothyronine With Gestational Hypertension, Preeclampsia, Preterm Birth, and Birth Weight: An Individual Participant Data Meta-analysis

**DOI:** 10.1210/clinem/dgad631

**Published:** 2023-10-25

**Authors:** Arash Derakhshan, Tuija Männistö, Liangmiao Chen, Joris A J Osinga, Ghalia Ashoor, Xuemian Lu, Sofie Bliddal, Fang-Biao Tao, Suzanne J Brown, Bijay Vaidya, Andrew T Hattersley, Sachiko Itoh, Polina V Popova, Ashraf Aminorroaya, Reiko Kishi, Maryam Kianpour, Elena A Vasukova, Abel López-Bermejo, Emily Oken, Leda Chatzi, Marina Vafeiadi, Wichor M Bramer, Judit Bassols, Aitana Lertxundi, Ana Fernández-Somoano, Paula Carrasco, Juha Auvinen, Kun Huang, Ulla Feldt-Rasmussen, Elena N Grineva, Erik K Alexander, Elizabeth N Pearce, Layal Chaker, John P Walsh, Robin P Peeters, Mònica Guxens, Eila Suvanto, Kypros H Nicolaides, Tim I M Korevaar

**Affiliations:** Department of Internal Medicine, Erasmus University Medical Center, Rotterdam 3015 GD, The Netherlands; Academic Center for Thyroid Diseases, Erasmus University Medical Center, Rotterdam 3015 GD, The Netherlands; Northern Finland Laboratory Center Nordlab and Medical Research Center Oulu, Oulu University Hospital and University of Oulu, Oulu 90570, Finland; Department of Endocrinology and Rui'an Center of the Chinese-American Research Institute for Diabetic Complications, Third Affiliated Hospital of Wenzhou Medical University, Wenzhou 325035, China; Department of Internal Medicine, Erasmus University Medical Center, Rotterdam 3015 GD, The Netherlands; Academic Center for Thyroid Diseases, Erasmus University Medical Center, Rotterdam 3015 GD, The Netherlands; Harris Birthright Research Center for Fetal Medicine, King’s College Hospital, London SE5 9RS, UK; Department of Endocrinology and Rui'an Center of the Chinese-American Research Institute for Diabetic Complications, Third Affiliated Hospital of Wenzhou Medical University, Wenzhou 325035, China; Department of Medical Endocrinology and Metabolism, Copenhagen University Hospital, Rigshospitalet, Copenhagen 2100, Denmark; Department of Clinical Medicine, Faculty of Health and Clinical Sciences, Copenhagen University, Copenhagen 1172, Denmark; Department of Maternal, Child and Adolescent Health, School of Public Health, Anhui Medical University, Hefei, Anhui 230032, China; Anhui Provincial Key Laboratory of Population Health & Aristogenics, Hefei, Anhui 230032, China; Department of Endocrinology and Diabetes, Sir Charles Gairdner Hospital, Nedlands 6009, Perth, Western Australia, Australia; Department of Endocrinology, Royal Devon University Hospital NHS Foundation Trust, University of Exeter Medical School, Exeter EX1 2LU, UK; Institute of Biomedical and Clinical Science, University of Exeter Medical School, Exeter EX1 2LU, UK; Center for Environmental and Health Sciences, Hokkaido University, Sapporo, Hokkaido 060-0808, Japan; World-Class Research Center for Personalized Medicine and institute of Endocrinology, Almazov National Medical Research Centre, Saint Petersburg 197341, Russia; Department of Internal Diseases and Endocrinology, St.Petersburg Pavlov State Medical University, Saint Petersburg 197341, Russian Federation; Isfahan Endocrine and Metabolism Research Center, Isfahan University of Medical Sciences, Isfahan 81745-33871, Iran; Center for Environmental and Health Sciences, Hokkaido University, Sapporo, Hokkaido 060-0808, Japan; Isfahan Endocrine and Metabolism Research Center, Isfahan University of Medical Sciences, Isfahan 81745-33871, Iran; World-Class Research Center for Personalized Medicine and institute of Endocrinology, Almazov National Medical Research Centre, Saint Petersburg 197341, Russia; Pediatric Endocrinology Research Group, Girona Biomedical Research Institute (IDIBGI) & Dr. Josep Trueta Hospital, Girona 17007, Spain; Departament de Ciències Mèdiques, Universitat de Girona, Girona 17007, Spain; Division of Chronic Disease Research Across the Lifecourse, Department of Population Medicine, Harvard Medical School, Boston, MA 02215, USA; Department of Population and Public Health Sciences UoSC, Keck School of Medicine, Los Angeles, CA 90033, USA; Department of Social Medicine, School of Medicine, University of Crete, Heraklion 700 13, Crete, Greece; Medical Library, Erasmus University Medical Centre, GD Rotterdam 3015, The Netherlands; Maternal-Fetal Metabolic Research Group, Girona Biomedical Research Institute (IDIBGI), Dr. Josep Trueta Hospital, Girona 17007, Spain; Spanish Consortium for Research on Epidemiology and Public Health (CIBERESP), Instituto de Salud Carlos III, Madrid 28029, Spain; Department of Preventive Medicine and Public Health, University of Basque Country, Leioa 48940, Spain; BIODONOSTIA Health Research Institute, San Sebastian 20014, Spain; Spanish Consortium for Research on Epidemiology and Public Health (CIBERESP), Instituto de Salud Carlos III, Madrid 28029, Spain; Unit of Molecular Cancer Epidemiology, University Institute of Oncology of the Principality of Asturias (IUOPA)–Department of Medicine, University of Oviedo, Oviedo 33006, Asturias, Spain; Institute of Health Research of the Principality of Asturias (ISPA), Oviedo 33006, Spain; Epidemiology and Environmental Health Joint Research Unit, FISABIO−Universitat Jaume I−Universitat de València, Valencia 46020, Spain; Department of Medicine, Universitat Jaume I, Castellón de la Plana 12071, Spain; Medical Research Center Oulu, Oulu University Hospital, and Center for Life Course Health Research, University of Oulu, Oulu 90570, Finland; Department of Maternal, Child and Adolescent Health, School of Public Health, Anhui Medical University, Anhui 230032, China; Scientific Research Center in Preventive Medicine, School of Public Health, Anhui Medical University, Anhui 230032, China; Department of Medical Endocrinology and Metabolism, Copenhagen University Hospital, Rigshospitalet, Copenhagen 2100, Denmark; Department of Clinical Medicine, Faculty of Health and Clinical Sciences, Copenhagen University, Copenhagen 1172, Denmark; World-Class Research Center for Personalized Medicine and institute of Endocrinology, Almazov National Medical Research Centre, Saint Petersburg 197341, Russia; Division of Endocrinology, Hypertension and Diabetes, Brigham and Women’s Hospital, Harvard Medical School, Boston, MA 02215, USA; Section of Endocrinology, Diabetes, and Nutrition, Boston University School of Medicine, Boston, MA 02215, USA; Department of Internal Medicine, Erasmus University Medical Center, Rotterdam 3015 GD, The Netherlands; Academic Center for Thyroid Diseases, Erasmus University Medical Center, Rotterdam 3015 GD, The Netherlands; Department of Endocrinology and Diabetes, Sir Charles Gairdner Hospital, Nedlands 6009, Perth, Western Australia, Australia; Medical School, University of Western Australia, Crawley, WA 6009, Australia; Department of Internal Medicine, Erasmus University Medical Center, Rotterdam 3015 GD, The Netherlands; Academic Center for Thyroid Diseases, Erasmus University Medical Center, Rotterdam 3015 GD, The Netherlands; Spanish Consortium for Research on Epidemiology and Public Health (CIBERESP), Instituto de Salud Carlos III, Madrid 28029, Spain; ISGlobal, Barcelona 08003, Spain; Pompeu Fabra University, Barcelona 08002, Spain; Department of Child and Adolescent Psychiatry/Psychology, Erasmus University Medical Centre–Sophia Children’s Hospital, GD Rotterdam 3012, The Netherlands; Department of Obstetrics and Gynecology and Medical Research Center Oulu, University of Oulu, Oulu 90570, Finland; Department of Women and Children’s Health, Faculty of Life Sciences and Medicine King’s College London, London WC2R 2LS, UK; Department of Internal Medicine, Erasmus University Medical Center, Rotterdam 3015 GD, The Netherlands; Academic Center for Thyroid Diseases, Erasmus University Medical Center, Rotterdam 3015 GD, The Netherlands

**Keywords:** triiodothyronine, pregnancy, preeclampsia, gestational hypertension, preterm birth, birth weight

## Abstract

**Context:**

Triiodothyronine (T3) is the bioactive form of thyroid hormone. In contrast to thyroid-stimulating hormone and free thyroxine, we lack knowledge on the association of gestational T3 with adverse obstetric outcomes.

**Objective:**

To investigate the associaiton of gestational free or total T3 (FT3 or TT3) with adverse obstetric outcomes.

**Methods:**

We collected individual participant data from prospective cohort studies on gestational FT3 or TT3, adverse obstetric outcomes (preeclampsia, gestational hypertension, preterm birth and very preterm birth, small for gestational age [SGA], and large for gestational age [LGA]), and potential confounders. We used mixed-effects regression models adjusting for potential confounders.

**Results:**

The final study population comprised 33 118 mother–child pairs of which 27 331 had data on FT3 and 16 164 on TT3. There was a U-shaped association of FT3 with preeclampsia (*P* = .0069) and a J-shaped association with the risk of gestational hypertension (*P* = .029). Higher TT3 was associated with a higher risk of gestational hypertension (OR per SD of TT3 1.20, 95% CI 1.08 to 1.33; *P* = .0007). A lower TT3 but not FT3 was associated with a higher risk of very preterm birth (OR 0.72, 95% CI 0.55 to 0.94; *P* = .018). TT3 but not FT3 was positively associated with birth weight (mean difference per 1 SD increase in TT3 12.8, 95% CI 6.5 to 19.1 g, *P* < .0001) but there was no association with SGA or LGA.

**Conclusion:**

This study provides new insights on the association of gestational FT3 and TT3 with major adverse pregnancy outcomes that form the basis for future studies required to elucidate the effects of thyroid function on pregnancy outcomes. Based on the current study, routine FT3 or TT3 measurements for the assessment of thyroid function during pregnancy do not seem to be of added value in the risk assessment for adverse outcomes.

Adequate maternal thyroid hormone availability during pregnancy is important for regulation of maternal metabolism, placentation, and fetal development (1). Consequently, abnormalities in thyroid function tests are a risk factor for adverse pregnancy and offspring outcomes, such as preeclampsia, preterm birth, and small for gestational age (SGA) neonates, that are leading causes for neonatal mortality, morbidity, and noncommunicable diseases in later life (2-4). In clinical practice, thyroid dysfunction during pregnancy is diagnosed based on abnormal thyroid-stimulating hormone (TSH) and free thyroxine (FT4) concentrations (5). While the association of TSH and FT4 with adverse pregnancy outcomes has been extensively studied, less is known about the association of free and total triiodothyronine (FT3 and TT3) concentrations with adverse pregnancy outcomes. However, filling this knowledge gap could add relevant new insights from different dimensions.

First of all, T3 is the bioactive form of thyroid hormone. Serum T3 concentrations are predominantly derived through intracellular conversion of T4 by deiodinase enzymes in peripheral tissues. Therefore, T3 is a different marker of tissue thyroid hormone action and physiology than TSH or FT4. Second, experimental studies show that T3 regulates placentation (6, 7) and T3 is transferred across the placental barrier, after which it regulates fetal growth and development; however, maternal T3 does not reach the fetal brain (8). Disruption of placentation and fetal growth are major risk factors for adverse pregnancy outcomes (9, 10). Finally, understanding the relation between T3 concentrations and adverse pregnancy and child outcomes are relevant in relation to levothyroxine treatment. Levothyroxine treatment outside of pregnancy usually decreases T3 concentrations and/or the ratio of T3 to T4, a change that theoretically could counteract any positive levothyroxine effects (11, 12).

A relevant side note is that the measurement of both FT3 and TT3 are subject to limitations (13). Out of the 2, FT3 is the biologically available hormone but immunoassays used to measure FT3 concentrations may have vulnerabilities related to the low concentrations and (protein) interference, as do measurements of FT4 (14). On the other hand, the measurement of TT3 concentrations is more reliable but largely reflects the nonavailable T3 and is considerably affected by pregnancy physiology, similar to that described for TT4 (14). It is likely that differences in biological and measurement characteristics also translate to differences in any added clinical value of measuring FT3 or TT3, but additional outcomes will have to be studied in order to fully understand such consequences, similar to what has been done for (F)T4 (15).

Investigating the association of free and total T3 with pregnancy outcomes can provide novel insights into the relation of maternal thyroid hormone availability and adverse pregnancy outcomes. Therefore, we investigated the association of FT3 and TT3 with adverse obstetric outcomes utilizing an individual participant data meta-analysis.

## Materials and Methods

The Consortium on Thyroid and Pregnancy (https://www.consortiumthyroidpregnancy.org) is a collaboration of birth cohorts that aims to study the association of maternal thyroid function and autoimmunity with adverse pregnancy and child outcomes (3). For the current study, we followed the Preferred Reporting Items for Systematic Reviews and Meta-Analyses for Individual Patient Data guidelines and preregistered our study protocol (PROSPERO 2019 CRD42019147955), and deviations from the protocol are shown elsewhere (Appendix p. 8 (16)). To identify studies for inclusion, we conducted a systematic literature search for publications on thyroid function during pregnancy, published from inception to June 11, 2020, with no language restrictions, using several databases (Medline [Ovid], Embase.com, Web-of-Science, Cochrane CENTRAL, and Google Scholar; detailed search terms and strategy are in the Appendix (16)). The search was repeated on June 21, 2022, to identify studies that would have been eligible for inclusion but were published after the original search. Also, we identified unpublished studies through personal contacts, advertisements at scientific conferences, and invitations to join the consortium in medical journals and on social media (17, 18). We included cohort studies in which data were collected prospectively and that consecutively included participants from the general population and/or without active selection based on health status with either FT3 or TT3 measurements together with data on gestational age at birth, birth weight, and/or hypertensive disorders of pregnancy (preeclampsia and/or pregnancy induced hypertension) available. We excluded studies in which participants received treatment based on (abnormal) thyroid function tests (predominantly hospital-based cohorts) or studies that only included women with (overt) thyroid disease. Possible studies for inclusion were independently assessed for suitability by 2 authors (T.I.M.K. and A.D.) and any disagreement was resolved by discussion with a third author (J.O.). Investigators from each eligible study were invited to join the consortium using the contact details on the identified reports; if unsuccessful, we used contact details of other published studies or contacted their coauthors or department. Upon agreement to participate, we collected individual participant data using a standardized codebook. All participants with either FT3 or TT3 measurements and outcome data available were included. We excluded participants with a miscarriage/stillbirth, preexisting thyroid disease or thyroid-interfering medication usage, in vitro fertilization treatment, or twin pregnancies. Quality of the studies and risk of bias were assessed using the Newcastle–Ottawa scale. All cohorts were approved by a local review board and required participant informed consent or had been granted exemption from it by the local ethics committee. More details on methods are provided elsewhere (Appendix (16)).

### Exposures

For all cohorts, concentrations of FT3 and TT3 were log-transformed and then converted to population-specific SD scores after removal of outliers (±4 SD from the mean) to enable comparison between different cohorts and assays.

### Outcomes

The primary outcomes were documented preeclampsia or gestational hypertension or the composite of these 2 outcomes (if both available in the cohort); preterm birth and very preterm birth (defined as a gestational age at birth of less than 37 or 32 weeks, respectively); and small for gestational age (SGA), large for gestational age (LGA), and birth weight (as a continuous variable). To define SGA and LGA, birth weight was standardized according to gestational age at birth and fetal sex per cohort to take into account population-specific variations. SGA was defined as a standardized birth weight below the 10th cohort-specific percentile, and LGA as a standardized birth weight above the 90th cohort-specific percentile, according to the definition of the World Health Organization (19). Secondary outcomes were gestational age at birth (as weeks continuously), low birth weight (birth weight below 2500 g), and macrosomia (birth weight above 4000 g).

### Statistical Analyses

We used generalized linear mixed-effect regression models with a random intercept for each cohort to study the association of FT3 and TT3 concentrations in full and normal range (2.5th to 97.5th percentiles per cohort) with preeclampsia, gestational hypertension, preterm birth, SGA, and LGA. Linear mixed-effect regression models with a random intercept for each cohort were used to study the association of FT3 and TT3 with birth weight or gestational age at birth. All analyses of primary outcomes were also performed by a 2-step approach with random-effect models according to DerSimonian and Laird to pool estimates of the cohorts and assess heterogeneity across studies using the I^2^ statistic and 95% CI (20). We assessed potential publication bias using funnel plots and Egger tests. All models were adjusted for maternal age, body mass index, ethnicity, smoking, parity, gestational age at blood sampling, fetal sex, and gestational age at birth (the last 2 just for birth weight outcomes). Risk differences and corresponding 95% CIs were calculated according to Newcombe and Bender, taking into account baseline risk imprecision calculated using the Wilson score method, and were adjusted for covariates and could thus deviate from nonadjusted percentages (21). We used multilevel multiple imputation for missing data on covariates creating 5 imputed datasets for pooled analyses (22). All statistical analyses were performed using R statistical software version 3.6.2 (R Development Core Team (2018), Vienna, Austria; packages *lme4*, *mice*, *micemd*, *metafor*, *sjPlot*).

### Sensitivity Analysis

We studied the highest and lowest 5th percentiles of FT3 and TT3 as additional parameters, as compared to the middle 50 percentiles to study the outskirts of the distributions while optimizing statistical power. Furthermore, we performed analyses for preterm and very preterm birth only in women with gestational age at the time of sampling earlier that 37 or 32 weeks, respectively. Also, analyses for preeclampsia and gestational hypertension were limited to women with gestational age at the time of sampling earlier than 24 weeks only. In addition, effect modification of the association of FT3 or TT3 with adverse pregnancy outcomes by known risk factors was investigated by adding product interaction terms to the models according to the predefined protocol. Finally, we additionally adjusted all analyses with FT4, TSH, or thyroid peroxidase antibodies (TPOAb) positivity to investigate independent associations of FT3 or TT3 with the risk of adverse obstetric outcomes.

## Results

**Figure 1.**
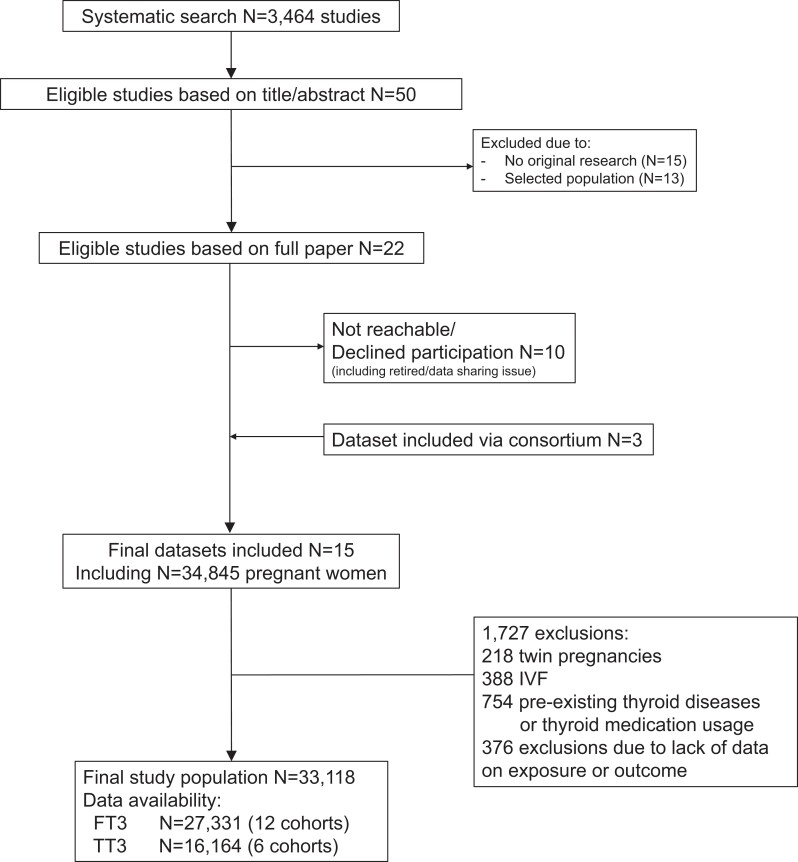
Flowchart of the study population.

We identified 3464 reports of which 50 were eligible for inclusion based on title and abstract screening (Fig. 1). After reading full texts, a total of 22 cohorts were invited to participate out of which 12 agreed to participate. Subsequently, after addition of 3 cohorts with unpublished data through members of the Consortium on Thyroid and Pregnancy, 15 cohorts were included in the study. We identified 4 studies after the search update that would have been eligible for inclusion but were published after the original search (Appendix p. 10 (16)). After exclusions, the final study population included 33 118 participants (Fig. 1) with a mean (SD) age of 29 (5.3) years and a median (95% range) gestational age at the time of sampling of 12.4 (7.0 to 40.0) weeks (Table 1). Cohort-specific characteristics, Newcastle–Ottawa Quality Assessment Scale scores, description of obstetric outcomes per cohort, and number (%) of missing data of covariates per cohort are provided elsewhere (Appendix (16)).

**Table 1.** Characteristics of the total study population (n = 33 118)*^α^*

Maternal characteristics	
**Age**, years	29.0 (5.3) (n = 33 078)
**Gestational age at the time of sampling**, weeks	12.4 (7.0 to 40.0) (n = 32 836)
Body mass index, kg/m^2^	23.5 (4.5) (n = 21 742)
**Parity**, n (%)	(n = 30 246)
0	17 671 (58.4)
1	9364 (31.0)
2	2100 (6.9)
≥3	1111 (3.7)
**Smoking status**, n (%)	(n = 32 259)
Non/past smoker	30 113 (93.3)
Current smoker	2146 (6.7)
**Maternal test results**	
**FT3** (pmol/L)	4.5 (2.8 to 6.4) (n = 26 973)
**TT3** (nmol/L)	1.7 (1.0. to 3.5) (n = 16 164)
**TPOAb positivity**, n (%)	2362/28 591 (8.3)
**Obstetric outcomes**	
**Preeclampsia**, n (%)	551/30 301 (1.8)
**Gestational hypertension**, n (%)	544/23 358 (2.3)
**Preterm birth (<37 weeks)**, n (%)	1827/33 022 (5.5)
**Very preterm birth (<32 weeks)**, n (%)	425/33 022 (1.3)
**Gestational age at birth**, weeks	39.6 (35.0 to 41.8) (n = 33 022)
**Birth weight, g**	3378 (529) (n = 32 454)
**Small for gestational age**, n (%)	3220/32 454 (9.9)
**Large for gestational age**, n (%)	3256/32 454 (10.0)

Table shows descriptive statistics of the characteristics of all included women as the mean (SD), median (95% range), or count (percentage), as appropriate. Cohort-specific descriptive characteristics are shown in the Appendix (16).

Abbreviations: FT3, free triiodothyronine; TT3, total triiodothyronine; TPOAb, thyroid peroxidase antibodies.

^*a*^Number of participants with available data on either FT3 or TT3 and any of the outcomes, unless otherwise indicated.

### Preeclampsia and Gestational Hypertension

**Figure 2.**
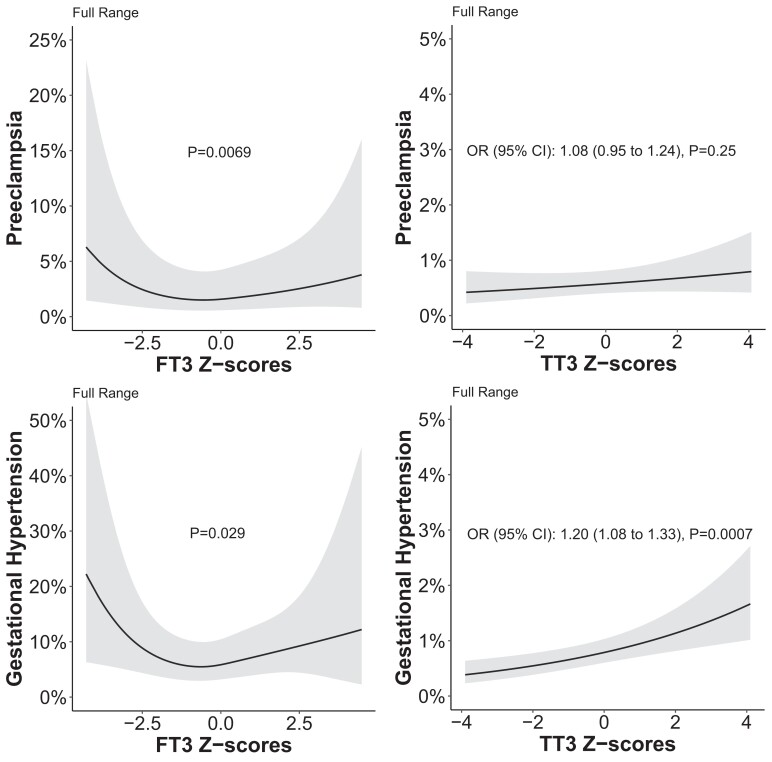
Association of full range FT3 and TT3 with preeclampsia and gestational hypertension.

There was a U-shaped association of FT3 with preeclampsia in both the full and normal range (Fig. 2) while there was no association of TT3 with preeclampsia. There was a U-shaped association of FT3 with gestational hypertension in the full range but there was no association in the normal range (Fig. 2; Figure 1 (16)); while a higher TT3 was associated with a higher risk of gestational hypertension in the full range but not in the normal range. The results for the composite outcome of hypertensive disorders of pregnancy were mainly driven by the associations of TT3 or FT3 with either preeclampsia or gestational hypertension (Figures 2 and 3 (16)). All results remained essentially unchanged when analyses were limited to women with a gestational age at the time of blood sampling earlier than 24 weeks (Figures 4 and 5 (16)). Further analysis showed that maternal age modifies the association of FT3 with gestational hypertension, with a higher FT3 being associated with a higher risk of gestational hypertension in women below 30 years of age (Figure 6 (16)).

### Preterm Birth

There was no association of FT3 with preterm birth or very preterm birth (Table 2). A higher TT3 was associated with a higher risk of preterm birth (OR 1.10, 95% CI 1.00 to 1.21; *P* = .039) in the normal range but not in the full range (Table 2). A higher TT3 was associated with a lower risk of very preterm birth in both the full and normal range (Table 2; OR 0.72, 95% CI 0.55 to 0.94 and 0.68, 0.49 to 0.94, respectively). There was no association of TT3 or FT3 with gestational age at birth (Table 9 (16)).

**Table 2.** The association of free and total T3 Z-scores with preterm birth

			Preterm birth			Very preterm birth	
	n	n (%) outcome	OR (95% CI)	*P* value	n (%) outcome	OR (95% CI)	*P* value
**Z-scores (full range)**							
**FT3**	26 510	1296 (4.9)	0.97 (0.91 to 1.03)	.36	169 (0.6)	0.86 (0.74 to 1.01)	.075
**TT3**	16 046	657 (4.1)	1.03 (0.95 to 1.12)	.35	53 (0.3)	0.72 (0.55 to 0.94)	.018
**Z-scores (normal range)**							
**FT3**	25 287	1218 (4.8)	1.00 (0.94 to 1.08)	.79	162 (0.6)	0.86 (0.71 to 1.04)	.12
**TT3**	15 279	626 (4.1)	1.10 (1.00 to 1.21)	.039	52 (0.3)	0.68 (0.49 to 0.94)	.020

Table shows the association of maternal free and total T3 (FT3 and TT3) in full or within the normal range (2.5th-97.5th percentiles) with preterm birth (<37 weeks) and very preterm birth (<32 weeks). All analyses were adjusted for maternal age, body mass index, ethnicity, smoking, parity, gestational age at blood sampling and fetal sex.

Abbreviations: FT3, free triiodothyronine; TT3, total triiodothyronine.

### Birth Weight

There was no association of FT3 or TT3 with SGA or LGA (Table 3). There was no association of FT3 with birth weight (Table 3), whereas each 1 SD higher TT3 was associated with 12.8 g (95% CI 6.5 to 19.1) higher mean birth weight in the full range and 15.0 g (7.4 to 22.5) in the normal range. There was no association of FT3 or TT3 with low birth weight or macrosomia (Table 10 (16)).

**Table 3.** The association of free and total T3 concentrations with birth weight

		Small for gestational age		Large for gestational age		Birth weight (g)	
	n	OR (95% CI)	*P* value	OR (95% CI)	*P* value	Mean difference (95% CI)	*P* value
**Z-scores (full range)**							
**FT3**	26 269	1.02 (0.97 to 1.06)	.34	1.00 (0.96 to 1.05)	.75	2.38 (−2.8 to 7.6)	.37
**TT3**	15 848	0.97 (0.91 to 1.01)	.22	1.03 (0.98 to 1.09)	.19	12.8 (6.5 to 19.1)	<.0001
**Z-scores (normal range)**							
**FT3**	25 059	1.00 (0.95 to 1.06)	.78	0.99 (0.94 to 1.05)	.88	3.87 (−2.4 to 10.1)	.22
**TT3**	15 091	0.94 (0.88 to 1.00)	.068	1.02 (0.96 to 1.09)	.48	15.0 (7.4 to 22.5)	.0001

Table shows the association of maternal FT3 and TT3 (Z-scores) in full or within the normal range (2.5th-97.5th percentiles) with small for gestational age (SGA), large for gestational age (LGA) and continuous birth weight (grams). All analyses were adjusted for maternal age, BMI, ethnicity, smoking, parity, gestational age at blood sampling and fetal sex and gestational age at birth (for birth weight only).

The sensitivity analyses of the association of lowest and highest 5th percentiles of TT3 and FT3 with the adverse obstetric outcomes (compared with the middle 50 percentiles) reflected the results of the continuous analyses (Tables 5 to 7 (16)). Additional adjustment with FT4, TSH, or TPOAb positivity did not change the results (Tables 11 and 12; Figures 7 to 9 (16)). Results of the 2-step analyses were generally the same as 1 step, and the Egger's test funnel plots did not show any significant publication bias (Figures 10 to 23 (16)). The I^2^ statistics for heterogeneity range from 0% to 59%, indicating low to moderate heterogeneity (Figures 10 to 23 (16)).

## Discussion

The relevance of gestational T3 measurements has remained unknown due to the historical focus on TSH and FT4 in this research field. To the best of our knowledge, this individual participant data meta-analysis is the largest study on the association of TT3 and FT3 with major adverse obstetric outcomes to date. We believe that the results of this study are best interpreted with a broad and critical view, taking into account especially the similarities and differences of the results for FT3 vs TT3 and considering the results from previous studies comparing the associations of TT4 vs FT4 with adverse pregnancy outcomes (14, 15).

We identified a U-shaped association of FT3 with gestational hypertension and preeclampsia while higher TT3 was associated with gestational hypertension but not with preeclampsia. These results are unlikely to have occurred due to reverse causation as analyses excluding measurements performed after 24 weeks showed similar results. Interestingly, in a previous study from this consortium (2), we showed that there is a U-shaped association of TSH but not FT4 with preeclampsia, which is a counterintuitive finding when considering the negative feedback system of the hypothalamic–pituitary–thyroid axis. The discrepancy between TSH and FT4 in that study may have been due to differences in FT3 concentrations. A counterargument for this is that there was no association of TSH or FT4 with gestational hypertension in our previous study, which is opposite to the current study. Future studies are required to investigate any potential disruption of thyroid function during preeclampsia following experimental studies that show disruption of transthyretin physiology and hepatic deiodinase activity (23, 24). This may indicate that current results and previous findings on FT4 and TSH could be spurious due to reverse causation. On the other hand, there is evidence that the effects of T3 on trophoblasts and maternal decidual cells partly regulates the imbalance of angiogenic factors and cytokines that play a major role in the etiology of adverse outcomes such as preeclampsia (25, 26). Therefore, we hypothesize that the findings related to hypertensive disorders in the current study more likely reflect pathologic effects of abnormal T3 availability on placentation rather than effects of hypertensive disorders of pregnancy on T3 availability. However, we cannot exclude the concept that early placental changes that would ultimately result in preeclampsia affect placental deiodinase expression or interfere in FT3 assays (27-29). Interestingly, additional adjustment with FT4, TSH, or TPOAb positivity did not change our results for the association of FT3 with hypertensive disorders of pregnancy which might mean the findings for FT3 are independent from effects by TSH or FT4.

In the current study, there was no consistent association of TT3 or FT3 with preterm birth (<37 weeks) which is similar to previous studies investigating FT4 and total T4 (3, 15). Interestingly, a lower TT3 was associated with a higher risk of very preterm birth (<32 weeks) and there was a similar direction for the association of FT3 with very preterm birth, although this association failed to reach statistical significance. This is in line with the results of a previous study from this consortium showing a negative association of FT4 with very preterm birth but not preterm birth (3). The difference between the results for preterm birth vs those for very preterm birth could be due to a different underlying etiology or it could be a reflection of the more overt phenotype of the same pathogenesis (30). Importantly, very preterm birth (<32 weeks) is an outcome that is clinically more relevant than preterm birth (<37 weeks). Large observational studies like the current study are the only practical means to study these detrimental clinical outcomes with a relatively low incidence considering the impracticality for randomized trials. The interpretation of this finding is complicated by the opposite result that a higher TT3 was associated with a higher risk of preterm birth, this could be a chance finding as it did not replicate in the full range and was a much smaller effect estimate than those for FT3 or very preterm birth. Nonetheless, we do aim to further replicate these findings and disentangle the current discrepancies using additional datasets in the future.

We could not identify an association of FT3 with birth weight outcomes while a higher TT3 was associated with a higher birth weight and a lower risk of SGA. A previous study has shown that TT3 was associated with higher fasting and postprandial glucose concentrations while higher FT4 was associated with lower fasting and postprandial glucose concentrations (31), which are established risk factors for increased fetal growth and birth weight. Therefore, the current discrepancy between TT3 and FT3 results for birth weight could be a reflection of feeding status. An alternative explanatory mechanism is that maternal binding protein status predominantly affects TT3 concentrations, which are a reflection of maternal estrogen concentration, which is positively associated with birth weight (32, 33). Future studies with repeated measurements and data on feeding status at blood draw are needed to further study these hypotheses.

The interpretation of any thyroid function test in pregnancy is complicated by changes in thyroid physiology and lack of available pregnancy and center-specific reference ranges for TSH and FT4. However, the interpretation of gestational FT3 and TT3 concentrations is further complicated by a paucity of data. FT3 could be the optimal reflection of potential T3 effects on the placenta and the fetus because it is a measurement of the biologically available T3, although it has been argued that the assays for FT3 do not perform as well as those for other thyroid function tests (34), which could be amplified during pregnancy. Nonetheless, the between-participant differences that are relevant for the current study are still likely to be interpretable and valid despite potential misclassification due to poor assay performance. While the TT3 assay has been less criticized on performance (34), the TT3 concentration reflects >99% of the biologically inactive hormone and changes considerably during pregnancy because of changes in thyroid hormone–binding globulins, predominantly thyroxine-binding globulin. Taking both the abovementioned considerations into account, as well as the results of this study, there seems to be no added value of routine FT3 or TT3 measurements during pregnancy, due to current knowledge gaps; however, the associations between FT3 and hypertensive outcome of pregnancy and between TT3 and birth weight warrant further study.

This is the first individual participant data meta-analysis investigating the association of both TT3 and FT3 with major adverse obstetric outcomes bringing together detailed datasets from prospective population-based cohorts around the world. Nonetheless, the results of this study should be considered as exploratory and form the basis for future studies assessing the effects of gestational thyroid function on pregnancy outcomes. Our study is limited because we were unable to include all cohorts with available data due to unwillingness to participate, data-sharing limitations, or lack of response from the principal investigator. It is also important to note that causality of the associations in this study cannot be inferred, and residual or unmeasured confounding cannot be excluded even though we have adjusted our models for several potential and known confounders.

## Conclusions

The results of this study provide novel insights on the association of gestational FT3 and TT3 with major adverse pregnancy outcomes, which can inform future studies and enhance the discussion on the relevance of FT3 vs TT3 measurements in pregnancy. However, when considering currently available knowledge including the results from our study, there seems to be no added value of routine FT3 or TT3 measurements during pregnancy.

## Data Availability

Restrictions apply to the availability of some or all data generated or analyzed during this study to preserve patient confidentiality or because they were used under license. The corresponding author will on request detail the restrictions and any conditions under which access to some data may be provided.

## Abbreviations

FT3: free triiodothyronine
FT4: free thyroxine
LGA: large for gestational age
SGA: small for gestational age
T3: triiodothyronine
TSH: thyroid-stimulating hormone
TT3: total triiodothyronine; TPOAb = thyroid peroxidase antibodies
